# Pharmacological Relaxation of the Ureter When Using Ureteral Access Sheaths during Ureterorenoscopy: A Randomized Feasibility Study in a Porcine Model

**DOI:** 10.1155/2016/8064648

**Published:** 2016-10-20

**Authors:** Søren Kissow Lildal, Kim Hovgaard Andreassen, Frederikke Eichner Christiansen, Helene Jung, Malene Roland Pedersen, Palle Jörn Sloth Osther

**Affiliations:** ^1^Urological Research Center, Department of Urology, Lillebælt Hospital, Fredericia, Denmark; ^2^Institute of Regional Health Research, University of Southern Denmark, Fredericia, Denmark

## Abstract

*Objective*. High intraluminal pressure during ureterorenoscopy (URS) increases risk of infectious and haemorrhagic complications. Intrarenal pressure may be reduced by the use of ureteral access sheaths (UASs), which on the other hand may cause ureteral damage. We have previously shown that the *β*-agonist isoproterenol (ISO), when administered topically in the irrigation fluid, is able to inhibit ureteral muscle tone and lower intrarenal pressure during URS. The aim of this study was to examine the effect of ISO on the success rate of UAS insertion in a porcine model.* Materials and Methods*. 22 pigs in which a UAS could not initially be placed were randomized to endoluminal irrigation with either ISO (0.1 *μ*g/mL) or saline before a new insertion trial. Subsequently, it was registered whether the UAS could be passed without resistance. During extraction of the sheath, any ureteral lesions were characterized ureteroscopically using the PULS classification system. Surgeons were blinded to randomization.* Results*. In the ISO group, the observed effect of irrigation was 63% successful UAS insertions, compared to 27% in the saline group. No serious lesions (<PULS grade 2) were observed in the ISO group.* Conclusions*. Endoluminal irrigation with ISO may facilitate UAS insertion and potentially decrease UAS related ureteral lesions.

## 1. Introduction

Ureteral access sheaths (UASs) are increasingly being used for ureterorenoscopic procedures (URS). The use of UAS has been shown to decrease intrarenal pressure during URS, thereby potentially reducing risk of septic and haemorrhagic complications [1, 2]. On the other hand, several recent reports have documented UAS usage to be associated with ureteral damage, which subsequently may result in stricture formation and loss of kidney function [3, 4]. Thus, UAS usage may be a double-edged sword: on the one hand diminishing risks related to irrigation and on the other hand increasing risks related to access in a narrow ureter [5]. However, the access related injuries might be related to not only the limited size of the ureter, but also the dynamics of the organ [4, 6]. We have previously shown that the *β*-agonist isoproterenol (ISO), when administered locally in a 0.1 *μ*g/mL saline solution at an irrigation rate of 8 mL/min, was able to inhibit the ureteral muscle tone and lower the pressure in the upper urinary tract significantly during ureterorenoscopy without causing systemic adverse effects [7–10].

We present a randomized feasibility trial of the effect of adding ISO to the irrigation fluid for successful UAS insertion in a porcine model.

## 2. Materials and Methods

The study was performed on 22 anaesthetized female pigs weighing 55 kg. The pigs were fed a standard diet during breeding. Before the study, they had access to water but were fasted 12 hours prior to anaesthesia.

After premedication with azaperone (4 mg/kg) and midazolam (4 mg/kg), anaesthesia was induced by propofol (4–20 mg/kg) and maintained with sevoflurane (1.2 MAC) and fentanil (0.03 mg/kg/h). The pigs were orotracheally intubated and mechanically ventilated (GE Healthcare S5 Avance). Hydration was maintained by administration of saline (9 g/L sodium chloride, 10 mL/kg/h) at a temperature of 37°C through an ear vein.

The 22 pigs were randomized into two groups each of 11 individuals. Each group was assigned to saline irrigation fluid as follows: ISO group, addition of 0.1 mg/mL ISO, or saline group, no ISO added. The investigators were blinded to randomization.

A cystoscope was inserted through the urethra into the bladder. A ureteral catheter (Selectip®, Bard Medical, Covington, USA) was placed in the distal part of the ureter and retrograde pyelography was performed to visualize the anatomy of the upper urinary tract. A hydrophilic guidewire (Sensor®, Boston Scientific, Marlborough, MA, USA) was placed via the ureteral catheter, through the ureter, to the renal pelvis, and the cystoscope was removed.

Over the guidewire an attempt of insertion of a UAS (Navigator™ Ureteral Access Sheath, Boston Scientific, Marlborough, MA, USA) 13/15 Fr. was made. The insertion was performed under fluoroscopic guidance. Advancement of the UAS was stopped, when the surgeon according to clinical experience subjectively felt that resistance was too great to proceed. The position of the tip of the UAS in the ureter was registered by fluoroscopy, and a distance mark was made on the sheath at the level of the external urethral meatus in order to locate the area of ureteral resistance. The UAS was removed and the distance mark was transferred to a dual lumen ureteral catheter 10 Fr. (Cook Medical, Bloomington, IN, USA), which was then inserted over the guidewire until the mark was at the level of the external urethral meatus and the tip of the catheter at the area of ureteral resistance.

The irrigation fluid was mounted on the second channel of the dual lumen catheter and administered with a pump at a constant irrigation rate of 8 mL/min for 5 minutes. Subsequently, a new attempt was made to insert the UAS, and it was registered if insertion proximal to the resistance was possible (= full effect) or not (= no effect).

Finally, a semirigid ureteroscope was inserted through the UAS, and during retraction of the sheath the ureter was inspected for visible lesions, which were characterized and registered using the PULS classification system [11].

In all pigs, UAS insertion was performed on the left ureter primarily. If no resistance was found, insertion was tried on the right ureter. If there was no resistance to the UAS on either side, ureteral dilators 16−18 Fr. (Boston Scientific, Marlborough, MA, USA) were inserted successively until resistance was met. If there was no resistance to these either, the pig was excluded from the study and replaced by a new pig that was assigned to the same treatment according to randomization.

**Figure 1:**
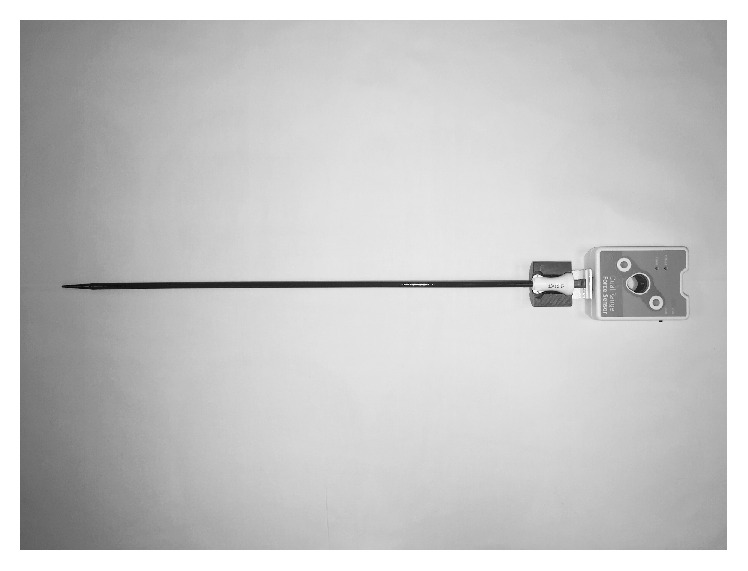
Ureteral access sheath 13/15 Fr. attached to the digital force meter.

**Figure 2:**
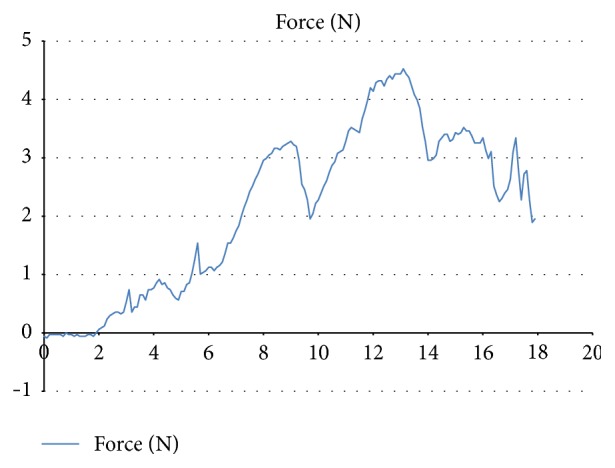
Force measurements registered 10 times/second displayed as graphic curves in MultiLab™ data analysis software. The highest force (*F*_max_) measured in newton (N) for more than 1 second was marked on each of the curves, defining *F*_max_ > 1 s as the maximum force applied to the UAS during insertion.

During every UAS insertion, measurements of the pushing forces were registered by a force meter (Fourier Force sensor) attached to the UAS and connected to a computer with recording software (NovaLink™ datalogger and MultiLab data analysis software) (Figures 1 and 2). The surgeon was blinded to the force measurement recordings. During retraction of UAS, the ureter was inspected with a ureteroscope; any ureteral lesions were evaluated and registered using the PULS classification system [11].

Finally, the pigs were euthanized under anaesthesia with 20 mL of pentobarbital, 200 mg/mL.

*Ethics*. The National Ethical Committee for Animal Experimentation (Copenhagen, Denmark) approved the study.

*Statistics*. The study was designed as two parallel single-arm studies [12]. The lowest acceptable rate of success was defined as 45% and the highest unacceptable success rate was defined as 10%. According to this, sample size was calculated as 11 in each arm. Within these definitions, observation of 4 or more full-effect insertions equals a success rate of at least 45%, whereas observation of less than 4 full-effect insertions means that the success rate may be below 10% [12]. Results were analysed with one-sample calculation of proportions in each group as two single-arm studies, using Stata software (StataCorp, Texas). Values are reported as estimates of proportions (95% CI).

## 3. Results

Normal upper urinary tract anatomy was found in all animals. In 5 cases, no resistance was found even to insertion of an 18 Fr. ureteral dilator, and these animals were excluded and replaced in order to obtain complete data from 22 individual pigs. In 6 of the tested 22 pigs, the ISO/NaCl experiment was completed using ureteral dilators due to no resistance to UAS 13/15 Fr. placement. According to randomization, ISO was administered in the irrigation fluid during 11 procedures and pure saline in 11.

In the ISO group, 7 of 11 UASs (64%, 95% CI: 35–92%) could be placed successfully, compared to 3 of 11 UASs (27%, 95% CI: 1–53%) in the saline group (*χ* ^2^ = 2.9; *p* = 0.8) (Table 1). Thus, despite being not statistically significant in this limited series, there was a trend towards a higher success rate in the ISO irrigation group.

Pushing-force measurement curves were recorded on all insertions of UAS 13/15 Fr. (Tables 2 and 3). The highest force (*F*_max_) measured for more than 1 second was marked on each of the curves, defining *F*_max_ > 1 s as the maximum effective force applied to the UAS during insertion (Figure 2). The mean *F*_max_ > 1 s in all preirrigation measurements was 5.34 N. The mean *F*_max_ > 1 s in cases of successful UAS insertion after ISO irrigation was 6.04 N. The mean *F*_max_ > 1 s in cases of successful UAS insertion after NaCl irrigation was 6.33 N. In comparison, the mean *F*_max_ > 1 s was 4.6 N in the 6 cases where there was no subjective resistance to UAS 13/15 Fr. insertion.

There were no statistically significant differences in mean *F*_max_ between groups.

All ureteral lesions were evaluated ureteroscopically according to the PULS classification scale (Tables 2 and 3). In 5 out of the 6 cases of successful UAS insertion after ISO irrigation, we observed a total of 4 grade 1 lesions and 6 grade 2 lesions. In the 3 cases of successful UAS insertion after NaCl irrigation, we observed a total of 4 grade 1 lesions and 1 grade 2 lesion.

In the 13 cases of unsuccessful secondary UAS insertion, there were 6 grade 1, 3 grade 2, and 2 grade 3 lesions found. Thus, the severity of lesions was lower in the ureters, in which the secondary insertion was possible.

## 4. Discussion

This randomized feasibility study suggests that placement of a UAS may be eased by use of endoluminal irrigation with the ureteral smooth muscle relaxant ISO, thus potentially decreasing UAS related ureteral injury and increasing efficacy of retrograde ureterorenoscopic procedures.

Globally, indications for flexible ureterorenoscopy (fURS) are currently expanding without high-level evidence (randomized controlled trials) to support its superiority to other treatment modalities [5]. In a lot of these procedures, UASs are being used, due to alleged advantages such as facilitating retrograde intrarenal access, lowering intrarenal pressure, protecting the scope, and expediting stone extraction [13]. On the other hand, the use of UAS, which incontrovertibly has to have a larger diameter than the scope itself, harbours a risk of damaging the ureteral wall, as has been demonstrated in several recent clinical series [3, 4, 6]. In these series, as well as in another recent series [14], it was shown that pre-JJ-stenting both increased efficacy of fURS and reduced complication rates related to access. This is probably due to the fact that prestenting suppresses the peristaltic mechanism, transforming the ureter into an adynamic tube that is much easier accessed with scopes and sheaths [5]. Thus, it is not just the size of the ureter that limits access; the dynamics of the organ (peristalsis) also influence access success. The fact that the second insertion trial in the present study was successful in some of the cases irrigated with saline may be explained by spontaneous changes in the peristalsis of the ureter in these cases. Current practice of today is to stent the ureter, if access is not possible at the primary procedure [14]. This is inconvenient and wasteful for the patient as well as the healthcare system, since a second procedure has to be scheduled. The present preliminary data suggest that ISO irrigation potentially may reduce the number of second procedures in more than 60% of cases. ISO in high concentrations may have cardiovascular side effects; however, we have in previous human trials shown that intraluminal irrigation with ISO in the very low concentration used in this trial (0.1 *μ*g/mL) did not result in measurable changes in pulse rate and blood pressure, nor could ISO be measured in venous samples [7, 8].

UAS placement in the present study was monitored with force measurements. The mean force needed to place the UASs was equivalent in the saline and ISO groups, documenting that the higher success rate in the ISO group was not due to the surgeon using more force for advancing the UAS. This is further documented by the fact that ureteral lesions (grades 1+2) were not more prevalent in this group. The use of the validated PULS grading system in this blinded, randomized trial adds to the strength of the observations. On the other hand, the main limitation of the study is that inability to place the UAS was subjectively judged by the surgeon. To some extent, the randomized design takes into account this limitation, and the fact that the forces needed to place/refrain from placement of the UASs were comparable to previously published force measurements in UAS insertion and semirigid ureteroscopy suggests that the subjective sensation of unacceptable ureteral resistance is a fair and universal parameter [15, 16]. The present study represents a preliminary feasibility study in a porcine model, and, before recommendation for clinical use, ISO for facilitating UAS usage has to be evaluated in a controlled clinical trial. The present data justify the notion that the methodology may be transferred to human clinical trials.

## 5. Conclusions

Endoluminal irrigation with ISO (0.1 *μ*g/mL) may facilitate UAS insertion and potentially decrease UAS related ureteral lesions.

## Figures and Tables

**Table 1:** Effect of irrigation with NaCl/ISO on successful UAS insertion in cases in which a UAS could not be placed initially.

Group	Effect	No effect	Total
NaCl	3 (27%)	8 (73%)	11 (100%)
ISO	7 (64%)	4 (36%)	11 (100%)

**Table 2:** Results of ISO irrigation group. *F*_max_ > 1 s = the highest force measured in newton (N) for more than 1 second. In the ureteral lesion column, more than one number indicates more than one lesion.

Study ID	UAS/dilator size (Fr.)	Effect (insertion)	*F*_max_ > 1 s before irrigation (N)	*F*_max_ > 1 s after irrigation (N)	Ureteral lesions (PULS grade)
2	15	Yes	2.4	5.0	0
4	15	Yes	5.4	5.2	1
9	18	Yes	7.8	N/A	1 + 1 + 2 + 2
10	18	Yes	4.4	N/A	2
11	18	No	4.3	N/A	2 + 3
12	15	Yes	6.0	5.6	2
15	15	No	5.6	6.9	1 + 1
16	15	Yes	3.8	4.4	0
17	15	No	4.9	6.6	0
21	15	No	6.5	5.2	1 + 3
22	15	Yes	5.4	8.3	2 + 2 + 1

**Table 3:** Results of NaCl irrigation group. *F*_max_ > 1 s = the highest force measured in newton (N) for more than 1 second. In the ureteral lesion column, more than one number indicates more than one lesion.

Study ID	UAS/dilator size (Fr.)	Effect (insertion)	*F*_max_ > 1 s before irrigation(N)	*F*_max_ > 1 s after irrigation (N)	Ureteral lesions (PULS grade)
1	15	No	4.9	6.1	0
3	15	No	6.2	7.2	3
5	15	Yes	6.4	7.0	1
6	18	No	4.7	N/A	2 + 2
7	15	No	4.8	5.7	0
8	15	Yes	5.3	7.0	2
13	15	No	5.6	5.1	0
14	15	Yes	4.7	4.9	1 + 1 + 1
18	18	No	3.3	N/A	1
19	18	No	3.2	N/A	1
20	15	No	7.6	9.8	1
